# Event and Comment

**Published:** 1909-12-15

**Authors:** 


### DENTAL REGISTER.

Vol. LXIII. December 15, 1909. No. 12.

### EVENT AND COMMENT.

**DENTAL WORK FOR SCHOOL CHILDREN.** The Rochester, N. Y., Sunday Herald of November 21st prints several pictures showing the facilities and work done by the Rochester Health Association for the poor school children of that city. Among them is a picture of the dental clinic, which is a nicely equipped operating room with modern facilities for the best operations. This clinic is taken care of by Dr. A. W. Smith who is in attendance every day from 2 to 5 P. M. Last year 4,506 operations were made, and all for children who would otherwise probably not have had any attention. This is certainly worth something and the people of Rochester are to be congratulated and the society responsible for carrying on so noble an institution should be most cordially commended and supported. This work is being done in many other places, Detroit, New York and Boston, and it promises much for the future health of the teeth of our people. It is possible, as has been predicted, that many of these efforts will not endure long enough to accomplish any permanent results, because the people who have means to assist in carrying on such benevolences too soon lose interest in them and withdraw their financial support and the cost is then thrown upon a few interested benevolent dentists who can't afford to sustain the clinics and it dies a natural death. This Rochester clinic however seems to have unusual persistence and has kept its work growing so fast and to such an urgent demand that it can't be stopped. This work ought to be placed on a basis of public support, and it could be, through the health officers, were it not for the fact that our modern politicians are not sufficiently grounded in benevolent activities to help on any organization of civic value unless there can be some chance of personal agrandizement hitched onto it for them. This Rochester experiment is of private origin and support, and therefore it is free from all forms of graft, and can not be exploited for a personal cause.

**DENTAL PROGRESS IN 1909.** That the year just closing has been one of stagnation or retrogression no reader of our current literature will believe. On the contrary a hasty sketch of the important events of the past twelve months will present an exhibit worthy of any progressive profession. There seems to have been an unusual activity in literary work. Our journals have been filled with excellent articles on almost every phase of the technical and scientific art of the profession. By a curious coincidence two new books of great value on the history of dentistry were published during the year. Two new works on dental therapeutics by eminent authors have appeared this year and two other standard works on the same subject have published new editions. We certainly should be up to date in this subject. No startling discovery in the art or science of the profession has appeared. The year has been on the art side used in adjusting the casting process to practicable methods, and some advances that promise improvement in technical methods are the result. It seems now that we shall be able to make cast metal plates of the higher fusing metals, such as aluminum and gold alloys. It has already been demonstrated that large bridges and partial plates of gold are easily practicable. Another year's experience with the silicious cements has made clear the value of this material in proper places and also demonstrated its unreliability in others, as well as the fact that the greatest skill is required in its manipulation to secure the best results. The greatest advancement of the year has probably been the general effort to encourage better oral hygiene by public agitation. Instruction in the public schools and free clinics for the poor seem to be the methods advocated. At the same time there seems to be a quickened conscience in private practice that promises well. The great congress at Berlin was the professional event of the year for the European, if not for the whole world.

**HONORS TO OUR GREAT MEN.** The Chicago Odontographic Society gives a grand dinner, in January, to Dr. G. V. Black and the Dentists of the East extend a similar honor to Dr. James Truman about the same date. Is it because these men are great in deeds, that we wish to honor them and so claim a share in the glory of their achievements? We can't think so. It is rather that we esteem them for the nobility of their character and because we so value this attribute in our own lives that we want to applaud in a public way the men who have achieved that for which we all aspire. It is the Divine in all our natures and we must give expression to our approval when it manifests itself in one of us more than another.

